# Supplementary material for: Virulence Factor Genes in Invasive Escherichia coli Are Associated with Clinical Outcomes and Disease Severity in Patients with Sepsis: A Prospective Observational Cohort Study
Source: Microorganisms. 2023 Jul 17;11(7):1827. doi: 10.3390/microorganisms11071827 (PMC10386379; doi:10.3390/microorganisms11071827)
Supplement: Supplementary file 1 [file microorganisms-11-01827-s001.zip › microorganisms-2441568-supplementary.pdf]

Supplementary information to "Virulence factor genes in invasive Escherichia coli are associated with clinical outcomes and disease severity in patients with sepsis: a prospective observational cohort study"

dr. Valentino D'Onofrio[1,2,3], dr. Reinoud Cartuyvels[4], dr. Peter Messiaen[1,2], dr. Ivan Barisic[5], Prof. dr. Inge Gyssens[1,3]

[1] Faculty of Medicine and Life Sciences, Hasselt University, Hasselt, Belgium

[2] Department of infectious diseases and immunity, Jessa hospital, Hasselt, Belgium

[3] Department of Internal Medicine and Radboud Center for Infectious Diseases, Radboud University Medical Center, Nijmegen, The Netherlands

[4] Department of Clinical Biology, Jessa hospital, Hasselt, Belgium

[5] Austrian Institute of Technology, Vienna, Austria

Table of contents

	Page
**Supplementary Table S1.** Prevalence of virulence factors in ExPEC according to the source	2
**Supplementary Table S2.** Literature search results	5
**Supplementary Figure S1.** Isolate characteristics. A: multilocus sequence type; B: serotype; C: plasmid types	7
**Supplementary Figure S2.** Virulence factor genes per isolate per MLST. The presence or absence of virulence factor genes is different between isolates within the same MLST. The isolates do not cluster per MLST.	8
**Supplementary Figure S3.** Rarefaction curve. The observed number of genes was plotted against the number of isolates. The solid line illustrates the observed species diversity, the dashed line the extrapolated diversity.	9
**Supplementary Figure S4.** Antibiotic resistance genes found in clinical isolates using the ResFinder database. Different colours represent resistance to different antibiotic classes.	10

Supplementary Table S1. Prevalence of virulence factors in ExPEC according to the source

DESIGNATION	ADHESIN	FUNCTION AND ASSOCIATED INFECTIONS	Feces (%)	Cystitis (%)	APN (%)	Blood (%)	LCS (%)
Type 1 fimbriae	*fimH*	Colonization factor and biofilm formation in EIs, especially cystitis and meningitidis, by binding receptor D-Mannose	68 to 99	62 to 100	88 to 99	90 to 98	92
Curli fimbriae	*csgAB*	Attachment and invasion of host cells, interaction with host proteins, activation of the immune system, bacterial aggregation and biofilm formation	34 to 36	44 to 100	24	53 to 92	NF
P fimbriae	*papGII*	Proinflammatory activity and colonization factor in EIs especially pyelonephritis by binding GbO4 and GbO3	17 to 23	13	64 to 67	48 to 68	20 to 50
	papGIII	Proinflammatory activity and colonization factor in EIs especially cystitis by binding receptors GbO5 and GloboA	8 to 9	22	19 to 35	14 to 27	1 to 6
S fimbriae	*sfaS*	Adhesion to intestinal and urinary tract cells and facilitate penetration into the tissues by binding receptors NeuNAc (α2-3)Gal in UTIs, meningitidis	7 to 32	13 to 34	88 to 99	39 to 97	28 to 59
F1C fimbriae	*focG*	Adhesion to epithelial and endothelial cells in the bladder and kidney	3 to 6	14 to 39	35	21	1 to 4
Auf fimbriae	*auf*	Auf fimbriae	21 to 27	67	NF	NF	62
RTX protein TosA	*tosA*	Adhesion to host cells derived from the upper urinary tract, enhances survival in disseminated infections and lethality during sepsis	11	16	29		
Dr fimbriae	*dra*	Binding to the receptors DAF of epithelial cells, type IV collagen and CEACAMs. Promotes host cells invasion	6	0 to 4	6 to 12	5	NF
Afimbrial adhesin	*afa*	Binding to receptor DAF on the epithelial cells and hemagglutination capacity	2 to 17	19 to 81	16	49	9
IrgA homolog adhesin	*iha*	Iron-regulated-gene-homolog adhesin	14 to 24	22	41	33 to 40	19
**DESIGNATION**	**INVASIN**	**FUCNTION AND ASSOCIATED INFECTIONS**	**Feces (%)**	**Cystitis (%)**	**APN (%)**	**Blood (%)**	**LCS (%)**
hemagglutinin	*hek/hra*	Promote autoaggregation, hemagglutination, epithelial cell invasion and is associated with bacteremia in newborns	14 to 28	28	33 to 66	45	11

Endothelial brain invasion	*ibeA*	Cell invasion into the host tissues such as endothelial cell by binding Vimentine and Caspr1 to promote penetration of the blood–brain barrier	1 to 15	13 to 23	29	4 to 11	32 to 38
**DESIGNATION**	**TOXINS**	**FUCNTION AND ASSOCIATED INFECTIONS**	**Feces (%)**	**Cystitis (%)**	**APN (%)**	**Blood (%)**	**LCS (%)**
α-hemolysin	hly	Pore-forming RTX toxin responsible for kidney injury and inflammation	4 to 15	15 to 43	38 to 47	34 to 77	9 to 30
Cytotoxic necrotizing factor 1	cnf1	activation of Rho GTPases, engaging in cell necrosis and promoting resistance to phagocytosis, inflammation, recurrent UTIs and blood-brain barrier penetration	3 to 13	13 to 39	28 to 47	17 to 49	6 to 27
Cytolethal distending toxin	cdt	DNAse inducing apoptosis and cellular senescence resulting in cell distention	0 to 1	9 to 17	12	0 to10	46
Colibactin	clb	PK-NRP compounds inducing DNA damage resulting preferentially in apoptosis in immune cells and premature cellular senescence in epithelial cells.	11 to 32	33	44	18 to 58	75
**DESIGNATION**	**Autotransporters**	**FUCNTION AND ASSOCIATED INFECTIONS**	**Feces (%)**	**Cystitis (%)**	**APN (%)**	**Blood (%)**	**LCS (%)**
Antigen43	*agn43*	Autotransporter involved in auto-aggregation, adhesion to host cells, biofilm development and persistence	41	29 to 94	65	69	NF
Secreted autotransporter toxin	Sat	Autotransporter having serine protease activity inducing cytotoxic effect and kidney injury	14 to 24	23 to 56	55 to 68	26 to 39	49
Vacuolating autotransporter toxin	Vat	Autotransporter having serine protease activity affecting mucins, facilitating epithelium colonization and inducing host cell vacuolization	0 to 49	42 to 68	44	12 to 71	51 to 76
Serin protease autotransporter	pic	Autotransporter exhibiting serine protease activity affecting mucins, facilitating epithelium colonization and damaging host cell membrane	0 to 23	21 to 34	14 to 40	0 to 36	NF
Contact-dependent growth inibition	cdiAB	Induce the growth inhibition of the bacterial target	NF	NF	NF	NF	NF
**DESIGNATION**	**IRON UPTAKE**	**FUNCTION AND ASSOCIATED INFECTIONS**	**Feces (%)**	**Cystitis (%)**	**APN (%)**	**Blood (%)**	**LCS (%)**
Enterobactin	ent, fepA	Acquisition of siderophore Enterobactin-Fe3 +	100	100	*	*	*
Aerobactin	aer, iuc, iut	Acquisition of siderophore Aerobactin-Fe ions in the host involved in UTI	16 to 41	24 ot 52	38 to 77	54 to 80	61 to 88

Salmochelin	iroN	Acquisition of siderophore Salmochelin-Fe ions in the host involved in UTI and meningitidis	5 to 26	33 to 74	76 to 78	44 to 63	38 to 75
Yersiniabactin	irp, fuyA	Acquisition of siderophore Yersiniabactin-Fe ions in the host involved in UTI and meningitidis	23 to 70	63 to 96	97	82	90 to 99
ChuA, Hma	chu, hma	Acquisition of Fe from hemoglobin in the host system	53 to 59	66 to 80	66 to 71	48 to 73	89
Sit	sitDCBA	Transportation of Fe and Mn	25	44	56	96	92
IreA	ireA	Iron-regulated homolog of siderophore receptors involved in UTI	7 to 19	13	47	32	NF
K1 capsule	*kpsMI-neuA*	Enable resistance to complement activation and phagocytosis, immunological tolerance and intracellular survival promoting both bacteremia and meningitis	0 to 27	35	13 to 47	8 to 82	75 to 90
TcpC	tcpC	Impairs the innate immune response by inhibiting Toll-like receptor and MyD88-specific signaling	8	22	42	32 to 39	0
D-serine deaminase	dsdCXA	Promotes bacterial growth by using D-serine as carbon, nitrogen, and energy source, and prevent the bacteriostatic activity of D-serine in the urine and brain.	0 to 4	20 to 22	NF	6	73 to 87
TrapT	traT	Inhibition of the classical pathway of complement activity and serum survival	43 to 88	36 to 68	49 to 81	50 to 81	NF
Increased Serum Survival	Iss	Resistance to complement and serum survival	4 to 20	13	35	10 to 34	25
Outer membrane protein T	OmpT	Resistance to protamine and urine survival	15 to 68	70	88 to 95	21 to 81	96

NF, data not found, *Present in almost all Escherichia coli genomes.

Supplementary Table S2. Literature search results

PubmedID	Authors, year, journal	Country	Objectives
**Original research**			
33235212	Biggel M et al., 2020, Nat Commun	Belgium, Hungary Denmerk, Sweden UK, USA	a genome-wide association study to investigate the population structure, virulence determinants, and evolution of invasive UPEC isolates, as compared to non-invasive UPEC isolates
32423949	Fröding I. et al, 2020, Antimicrob Agents Chemother	Sweden	To study the molecular epidemiology of community-onset bloodstream infection caused by ESBL/pAmpC-EC
22571989	Skjøt-Rasmussen L et al., 2012, Int J Med Microbiol	Denmark	To study *E. coli* isolates from patients with bacteraemia of urinary tract origin according to virulence-associated genes, phylogroups, and antimicrobial resistance, and the relation of these factors to hospital- vs. community-acquired origin, sex, and mortality.
31076319	Hung WT et al. 2019, J Microbiol Immunol Infect	Taiwan	To study whether specific sequence type (ST) and VFs of extended-spectrum beta-lactamase-producing E. coli (ESBL-EC) are associated with different outcomes in patients with bloodstream infection.
31245301	Daga AP et al. 2019, Front Cell Infect Microbiol	Brazil	to characterize the VFs, antimicrobial sensitivity profile, and epidemiological data of E. coli isolates from the blood of patients
23588104	Rodríguez-Baño J et al., 2013, J Infect	Spain	To investigate the impact of VF and other microbiological determinants on the outcome of patients with invasive infections due to extended-spectrum β-lactamase-producing Escherichia coli
26517481	Cyoia PS et al., 2015, J Infect Dev Ctries	Brazil	To analyze the presence of VF encoding genes, PAI sequences and phylogenetic groups of ExPEC strains isolated from urine and blood of patients compare them with 50 faecal commensal strains from healthy individuals.
33362261	Paramita RI et al., 2020 Plos One	Indonesia	to characterize the serotypes, MLST, virulence genes, and antimicrobial resistance of E. coli isolated from bloodstream infection patients
23801304	Szemiako K et al., 2013, Eur J Clin Microbiol Infect Dis	Poland	To analyze the phylogenetic distribution and virulence genes of Escherichia coli isolates which predispose bacteria to translocate from the urinary tract to the bloodstream

25654604	Mora-Rillo M et al., 2015, Virulence	Spain	To study the characteristics of *E. coli* causing bacteremia and their relationship with sepsis severity and mortality.
**Reviews**			
33101219	Desvaux M et al., 2020, Front Microbiol	N.A.	To summarize the current knowledge on the structure and functional features of PAIs, on PAI-encoded E. coli pathogenicity factors and on the role of PAIs in host–pathogen interactions

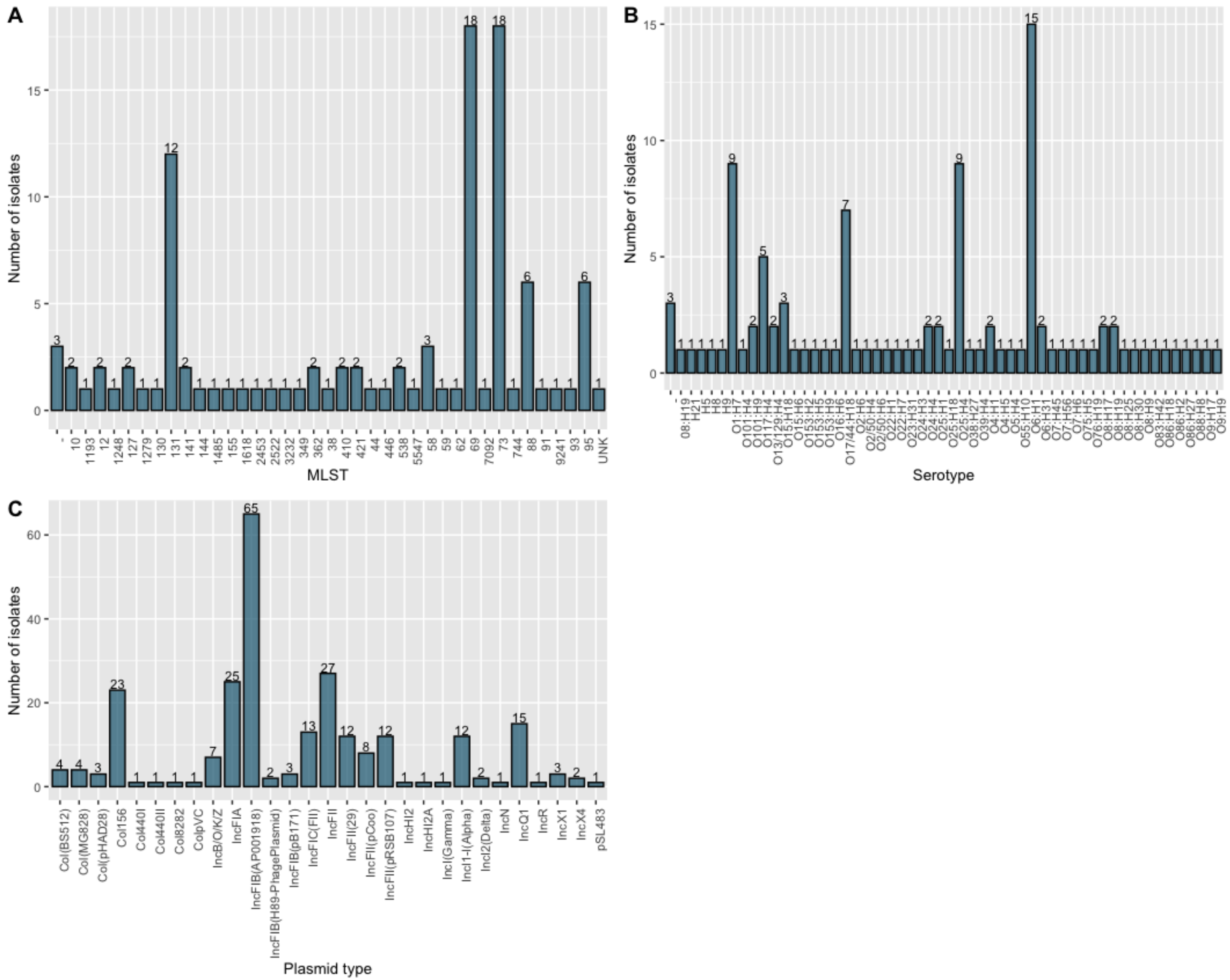

Supplementary Figure S1. Isolate characteristics. A: multilocus sequence type; B: serotype; C: plasmid types

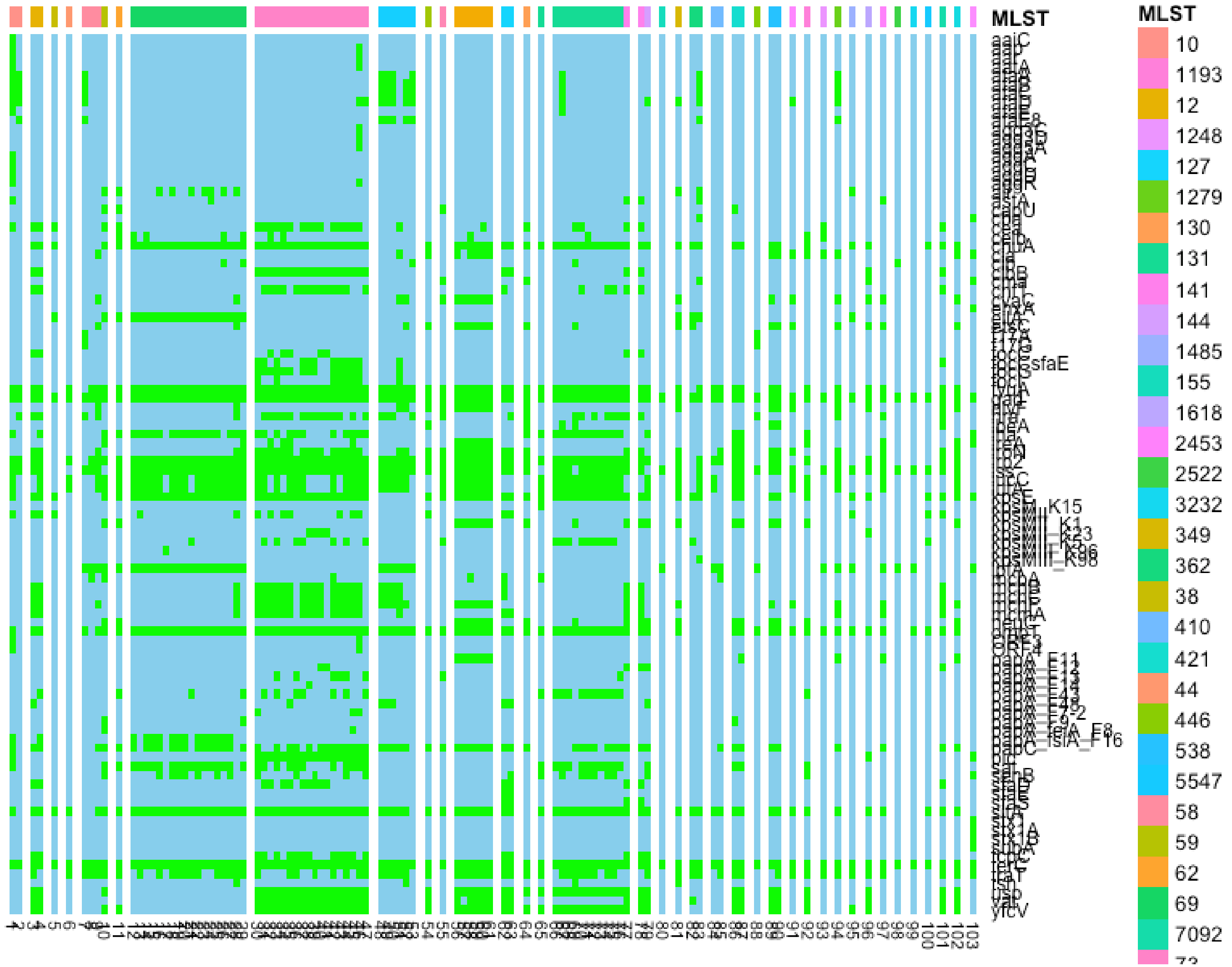

Supplementary Figure S2. Virulence factor genes per isolate per MLST. The presence or absence of virulence factor genes is different between isolates within the same MLST. The isolates do not cluster per MLST.

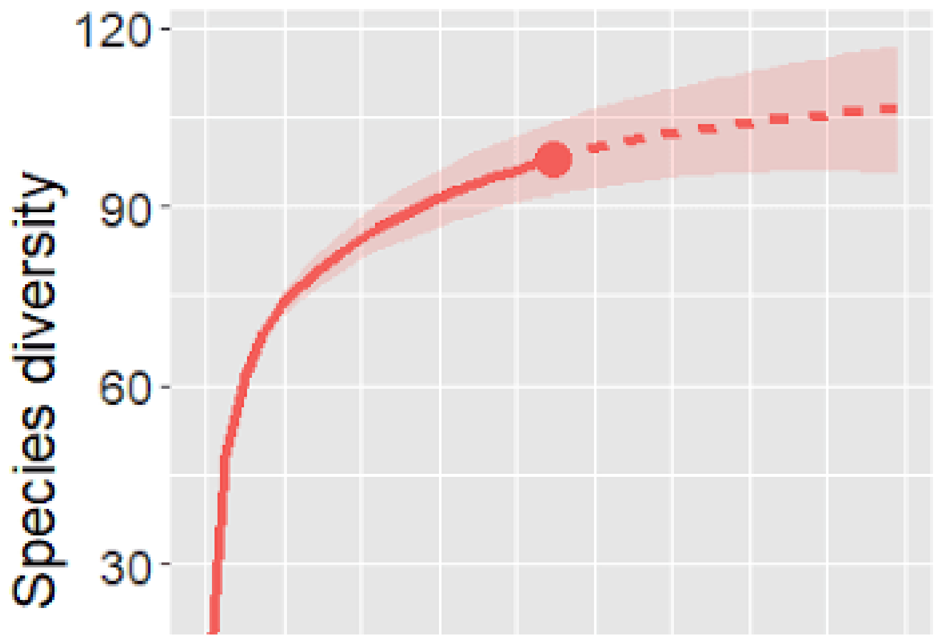

Supplementary Figure S3. Rarefaction curve. The observed number of genes was plotted against the number of isolates. The solid line illustrates the observed species diversity, the dashed line the extrapolated diversity.

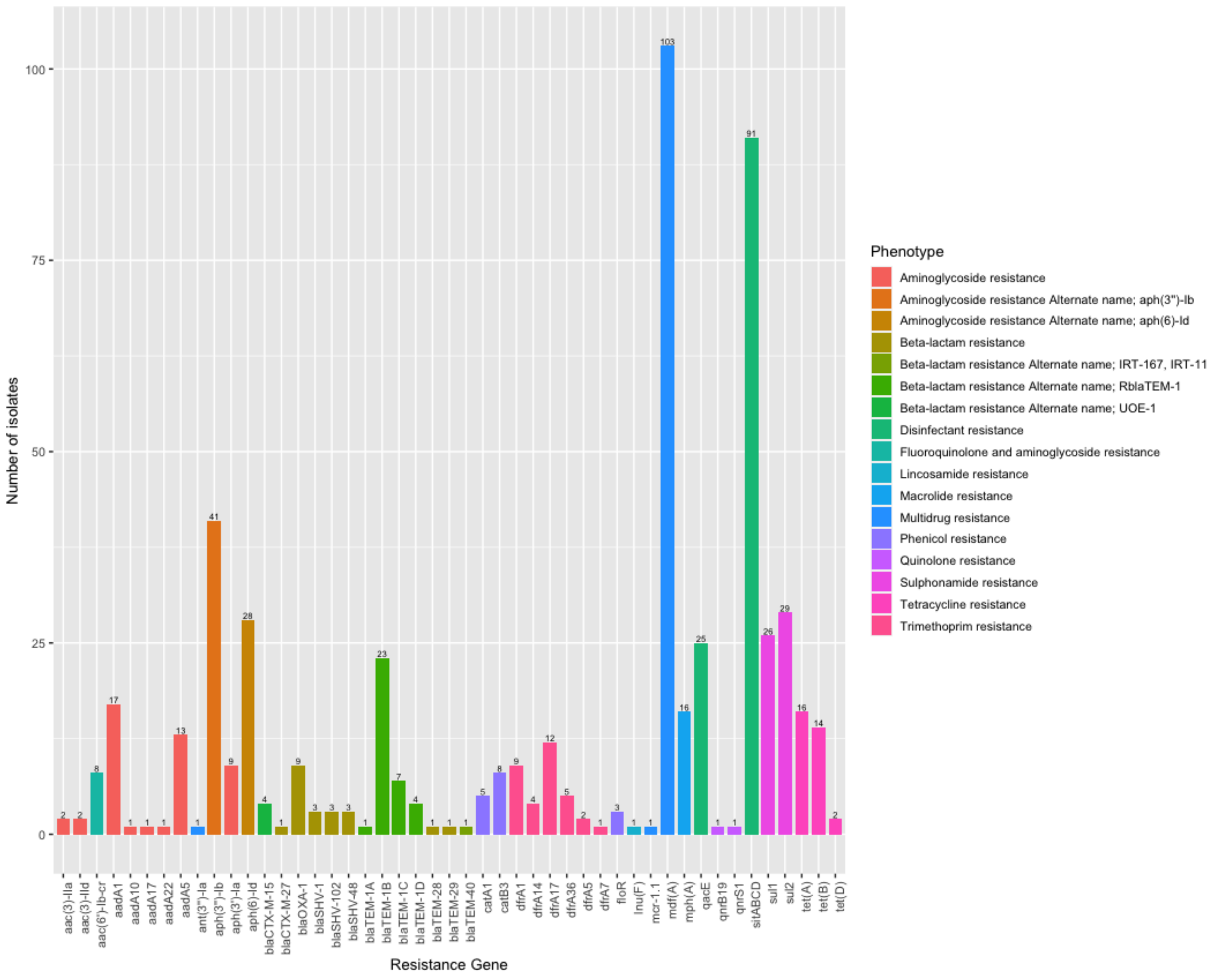

Supplementary Figure S4. Antibiotic resistance genes found in clinical isolates using the ResFinder database. Different colours represent resistance to different antibiotic classes.
